# A Case of Immune Thrombocytopenia After COVID-19 Infection

**DOI:** 10.7759/cureus.15843

**Published:** 2021-06-22

**Authors:** Gauthier Stepman, Ivy Daley, Duncan Bralts, Jigneshkumar B Patel, Johnathan Frunzi

**Affiliations:** 1 Internal Medicine, Medical Center of Trinity, Trinity, USA; 2 Gastroenterology, Medical Center of Trinity, Trinity, USA

**Keywords:** covid-19, immune thrombocytopenia, thrombocytopenia, petechiae, hematology

## Abstract

Immune thrombocytopenia (ITP) is a hematological condition that is characterized by a low platelet count. ITP can be primary or secondary. Secondary causes are diverse and include viral infections. The novel coronavirus has rarely been recognized as cause of ITP. This is a case of an 82-year-old Caucasian male who was infected by the novel coronavirus four weeks prior. His platelet count on admission was 1,000/mm^3^. He was diagnosed with ITP caused by the novel coronavirus as there were no other causes for his thrombocytopenia. The patient was treated with platelet infusions, high-dose corticosteroids, and intravenous immunoglobulin infusions.

## Introduction

Immune thrombocytopenia (ITP) is an autoimmune condition in which the patient’s platelets are destroyed by autoantibodies [1]. A platelet count of less than 100,000/mm3 is necessary for diagnosis [1]. The etiology for ITP is diverse and includes, but is not limited to, primary ITP in which the exact cause is unknown - drug-induced, lymphoproliferative disorders, immunodeficiency syndromes, post-infection (mainly viral), and other autoimmune diseases [1]. The presentation of ITP is diverse, ranging from asymptomatic thrombocytopenia to mild mucocutaneous bleeding or severe, life-threatening blood loss [1]. ITP is also associated with an increased risk of venous thromboembolism [2]. SARS-CoV-2 (COVID-19) has rarely been associated with thrombocytopenia [2-14]. The treatment of ITP consists of platelet transfusions, glucocorticoids, and intravenous immunoglobulin (IVIG) infusions [1].

## Case presentation

We present the case of an 82-year-old Caucasian male who presented to the hospital with a near-syncopal episode. The patient also complained of dyspnea on exertion for the past few weeks. He has a past medical history significant for hypertension, hypothyroidism, type 2 diabetes mellitus, and coronary artery disease status post coronary artery bypass grafting. The patient was diagnosed with COVID-19 four weeks prior to presentation to the hospital. He was treated with remdesivir and low dose dexamethasone. He was discharged after a three-day hospital stay. He noticed epistaxis the night prior to the presentation. He had also noted diffuse bruising on his arms several days prior to his current presentation. He denied a history of hematologic disorders.

On admission, his vital signs showed a temperature of 98.7 F, pulse rate of 82 beats per minute, respiratory rate of 17 breaths per min, blood pressure of 141/63 mmHg, and oxygen saturation of 89% on room air.

Physical examination revealed large areas of ecchymosis on the upper extremities, scattered ecchymosis on his lower extremities, some dried blood in his nares, and left lower lobe rales and rhonchi. Laboratory values yielded a white blood cell count 7,430/mm^3^, hemoglobin 10.3 g/dL, platelet count 1,000/mm^3^, prothrombin time 10.0 seconds, international normalized ratio 1.01, activated partial thromboplastin time 29 seconds, sodium 132 mmol/L, potassium 4.2 mmol/L, chloride 99 mmol/L, carbon dioxide of 25 mmol/L, blood urea nitrogen 45 mg/dL, and creatinine 1.4 mg/dL.

The patient was admitted to the medical floor. Hematology was consulted for severe thrombocytopenia.

**Figure 1:**
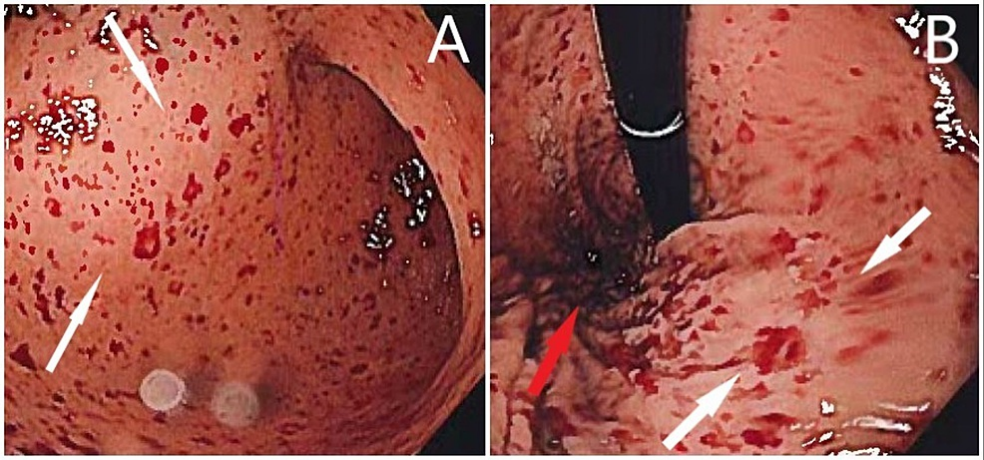
(A) Petechiae (white arrows) in the gastric antrum. (B) Petechiae (white arrows) in the gastric fundus (red arrow).

On day 3 of his hospitalization, he started complaining of melena. His hemoglobin count dropped to a low of 6.8 g/dL on hospital day 4. One unit of packed red blood cells (pRBC) was transfused, which resulted in an increase of his hemoglobin to 8.0 g/dL. Gastroenterology was consulted to further evaluate. An esophagogastroduodenoscopy was performed, which revealed mild-to-moderate diffuse gastritis. Diffuse petechiae were also noted in the entire stomach (Figures 1A, 1B).

The patient received four units of platelets without a satisfactory increase in his platelet count. The patient was then started on therapy with IVIG. He received a total of four doses of 0.4 g/kg of IVIG and was started on high-dose intravenous dexamethasone. This regimen raised his platelet count from 1,000/mm^3^ prior to administration to 161,000/mm^3^ after four doses (Table 1). His hemoglobin count also remained stable with the normalization of his platelets.

**Table 1:** Platelet count of our patient per day with associated intravenous immunoglobulin dose IVIG - Intravenous immunoglobulin

Hospital day	Platelet count	IVIG dose*
1	1,000/mm^3^	0
2	0/mm^3^	0
3	1,000/mm^3^	0
4	4,000/mm^3^	1
5	36,000/mm^3^	3
6	121,000/mm^3^	4
7	161,000/mm^3^	4

The patient was discharged with a 14-day course of 60 mg prednisone daily. The patient was seen four weeks later in the hematology-oncology clinic and was faring well with no recurrent bleeding or thrombocytopenia.

## Discussion

Patients diagnosed with COVID-19 infection may develop ITP. ITP can be primary or secondary, in which case preceding viral infections often are the culprit [1]. In a landmark 2020 systematic review to analyze the clinical profile and outcomes of new-onset ITP in COVID-19 patients, Bhattacharjee and Banerjee reported that ITP can occur following COVID-19 infection [14]. We report a patient who presented with a platelet count of 1,000/mm^3^. He denied taking any medications that frequently cause ITP, but did report a COVID-19 infection three weeks prior to presentation. Only a few case reports have been reported showing COVID-19 as the cause of ITP [2-14]. Most of the case reports published at this time in the peer-reviewed literature report patients who tested positive at presentation [2-14]. Of the 45 patients identified by Bhattacharjee and Bannerjee, only one patient tested negative for COVID-19 [14]. This patient was reported by Chen et al. [12]. However, this patient only tested negative on rapid nucleic acid amplification testing [12]. Our patient was different from this case as he tested negative on both rapid nucleic acid amplification testing as well as the more sensitive polymerase chain reaction testing.

Treatment modalities for COVID-19-induced ITP consist of treating COVID-19 with antiviral medication (such as Remdesivir) and supportive care if the patient tests positive, platelet infusions, high-dose glucocorticoids, and IVIG if glucocorticoids are ineffective [1]. Our patient did not need treatment for COVID-19 as he tested negative. Platelet infusions were ineffective, which can potentially be explained by the autoantibodies against platelets. He did respond to treatment with both glucocorticoids and IVIG. Notably as illustrated in Table 2, most patients respond well to either IVIG or corticosteroids, or a combination of both [2-13].

**Table 2:** Comparison of case reports previously published IVIG - Intravenous immunoglobulin

Authors	Number of patients	COVID-19 status in hospital	Lowest platelet count (x10^3^/mm^3^)	Treatment
Bomhof et al., 2020 [2]	3	Patient 1: Positive; Patient 2: Positive; Patient 3: Positive	Patient 1: <3; Patient 2: 2; Patient 3: 3	Patient 1: Glucocorticoids; Patient 2: Glucocorticoids+IVIG; Patient 3: Platelet transfusion
Bennett et al., 2020 [3]	1	Positive	<3	Platelet transfusion, IVIG
Hindilerden et al., 2020 [4]	1	Positive	10	IVIG, glucocorticoids
Pedroso et al., 2020 [5]	2	Patient 1: Positive; Patient 2: Positive	Patient 1: 2; Patient 2: 38	Patient 1: Platelet transfusion, glucocorticoids; Patient 2: No specific treatment
Lobos et al., 2020 [6]	1	Positive	1	IVIG
Artru et al., 2020 [7]	1	Positive	1	IVIG, glucocorticoids
Lorenzo-Villalba et al., 2020 [8]	3	Patient 1: Positive; Patient 2: Positive; Patient 3: Positive	Patient 1: 1; Patient 2: 2; Patient 3: 46	Patient 1: IVIG; Patient 2: IVIG; Patient 3: No specific treatment
Merli et al., 2020 [9]	1	Positive	6	IVIG, glucocorticoids
Murt et al., 2020 [10]	1	Positive	9	Glucocorticoids, IVIG
Sadr et al., 2020 [11]	1	Positive	16	Not reported
Chen et al., 2020 [12]	1	Negative	2	IVIG, glucocorticoids
Humbert et al., 2020 [13]	1	Positive	4	Glucocorticoids, IVIG

Although COVID-19 is a viral infection that mostly causes respiratory symptoms, the presentation of ITP as seen in our patient is diverse and can range from asymptomatic presentation to severe, life-threatening bleeding. Our patient had an episode of epistaxis that resolved but subsequently developed an upper gastrointestinal tract bleed. The treatment of active bleeding in ITP is similar to the treatment of the ITP itself, including platelet transfusions, glucocorticoids, and IVIG [1].

## Conclusions

ITP is a potentially life-threatening autoimmune condition that needs prompt diagnosis and treatment that might lead to better outcomes for patients. COVID-19 needs to be recognized as a secondary cause for ITP. Healthcare providers should be mindful of ITP in COVID-19 patients during the active infection and after they have recovered from the illness.
